# Supplementary material for: Clinical Utility of Plasma Cell-Free DNA EGFR Mutation Analysis in Treatment-Naïve Stage IV Non-Small Cell Lung Cancer Patients
Source: J Clin Med. 2022 Feb 21;11(4):1144. doi: 10.3390/jcm11041144 (PMC8880481; doi:10.3390/jcm11041144)
Supplement: Supplementary file 1 [file jcm-11-01144-s001.zip › jcm-1597614-supplementary.pdf]

Supplementary Table S1. Diagnostic performance of plasma and tissue EGFR mutation analysis (N = 311)

Variables	Plasma EGFR MUTATION Test		Tissue EGFR Mutation Test		*p*-Value
	**n/N**	**% (95% CI)**	**n/N**	**% (95% CI)**	
Sensitivity	132/164	80.5 (73.6–86.3)	130/164	79.3 (72.3–85.2)	0.787
Specificity	147/147	100.0 (97.5–100.0)	147/147	100.0 (97.5–100.0)	-
Accuracy	279/311	89.7 (85.8–92.9)	277/311	89.1 (76.2–85.4)	0.808
PPV	132/132	100.0	130/130	100.0	-
NPV	147/179	82.1 (77.1–86.2)	147/181	81.2 (76.2–85.4)	0.826

EGFR, epidermal growth factor receptor; CI, confidence interval; PPV, positive predictive value; NPV, negative predictive value.
